# Genetic Parameters of Different FTIR-Enabled Phenotyping Tools Derived from Milk Fatty Acid Profile for Reducing Enteric Methane Emissions in Dairy Cattle

**DOI:** 10.3390/ani10091654

**Published:** 2020-09-15

**Authors:** Giovanni Bittante, Claudio Cipolat-Gotet, Alessio Cecchinato

**Affiliations:** 1 Department of Agronomy, Food, Natural Resources, Animals and Environment (DAFNAE), University of Padova, Viale dell’Università 1, 35020 Legnaro, Italy; bittante@unipd.it; 2 Department of Veterinary Science, University of Parma, Via del Taglio 10, 43126 Parma, Italy; claudio.cipolatgotet@unipr.it

**Keywords:** mid-infrared (MIR) spectra, genetic parameters, ecological footprint, greenhouse gases, global warming

## Abstract

**Simple Summary:**

Enteric methane emission (EME) in dairy cows can feasibly be mitigated through genetic improvement at the population level. This work shows that several EME-related traits, directly and indirectly predicted from infrared spectra of milk, are heritable and are genetically correlated with those based on the fatty acid profile of milk. Genetic parameters were estimated using univariate and bivariate animal models. The results show that easy-to-measure values correlated to EME traits were identified and seem to have the potential to be exploited in breeding programs to improve the impact of dairy farming on climate change.

**Abstract:**

This study aimed to infer the genetic parameters of five enteric methane emissions (EME) predicted from milk infrared spectra (13 models). The reference values were estimated from milk fatty acid profiles (chromatography), individual model-cheese, and daily milk yield of 1158 Brown Swiss cows (85 farms). Genetic parameters were estimated, under a Bayesian framework, for EME reference traits and their infrared predictions. Heritability of predicted EME traits were similar to EME reference values for methane yield (CH_4_/DM: 0.232–0.317) and methane intensity per kg of corrected milk (CH_4_/CM: 0.177–0.279), smaller per kg cheese solids (CH_4_/SO: 0.093–0.165), but greater per kg fresh cheese (CH_4_/CU: 0.203–0.267) and for methane production (dCH_4_: 0.195–0.232). We found good additive genetic correlations between infrared-predicted methane intensities and the reference values (0.73 to 0.93), less favorable values for CH_4_/DM (0.45–0.60), and very variable for dCH_4_ according to the prediction method (0.22 to 0.98). Easy-to-measure milk infrared-predicted EME traits, particularly CH_4_/CM, CH_4_/CU and dCH_4_, could be considered in breeding programs aimed at the improvement of milk ecological footprint.

## 1. Introduction

Ruminants seem to be responsible for up to 18% of global greenhouse gas emissions [1,2], and dairy cows produce a significant proportion of those emissions. In its efforts to mitigate the effects on climate change of enteric methane emissions (EME) from dairy cattle populations, the dairy industry is looking at strategies for improving the feeding efficiency, but also at the genetic selection of animals [3], also because demand for milk and dairy products is increasing at world level. Although EME traits are governed by microbial activity in the rumen, they have also been shown to be dependent on the animal’s genetics, leading to the concept of indirect heritability [4].

Direct quantification of greenhouse gases (GHG) in respiration chambers (the gold standard in EME analysis) is not feasible individually for a large number of lactating cows. Moreover, respiration chamber environment and management are very different from those of commercial dairy farms. Predictions of EME at the farm level using detectors placed within automatic milking or feeder systems to analyze air methane and carbon dioxide concentrations is also problematic: they do not measure the daily volume of methane emitted and, despite having been proposed as a method for selecting dairy cows [5], they have shown a low reproducibility and correlation with respiration chambers and other methods [6,7,8], opening a wide debate in the scientific community [9,10,11].

As reviewed by Negussie et al. [12], the analysis of milk fatty acid (FA) profiles, along with the proper combination of FAs, is also considered an effective, promising method for predicting GHG, which van Gastelen and Dijkstra [13] have suggested for use in field conditions. It exploits the physiological links between EME, the type of fermentation in the rumen, and the proportions of volatile FAs produced in the rumen, absorbed by the intestines and used for de novo synthesis of FAs in the udder [14,15]. Combining data obtained through a meta-analysis of the results from eight experiments carried out in respiration chambers and taking account of 30 different diets, van Lingen et al. [16] devised two equations: one for predicting methane yield per unit of DMI (CH_4_/DM, g/kg), and one for predicting methane intensity per unit of fat/protein-corrected milk (CH_4_/CM, g/kg). The R^2^ from these equations was, respectively, 0.54 and 0.47, lower than accuracy found in some studies carried out on single experiments, but acceptable considering that it has been produced from a meta-analysis, so much more representative in terms of sources of variation included (countries, diets, research centers, years, cows and replicates). In a previous study [17], we used these equations to identify the sources of variation (dairy system, herd, parity and lactation stage) in these traits estimated from a large survey of 1158 Brown Swiss cows from 85 herds; we assessed methane production per cow (dCH_4_, g/d) by multiplying CH_4_/CM by the daily corrected milk yield (dCMY, kg/d), and associated these data to the cheese yield of individual cows to estimate methane intensity per unit of fresh cheese (CH_4_/CY_CURD_, g/kg) and per unit of cheese solids (CH_4_/CY_SOLIDS_, g/kg). Even though the breed of cows and the environmental conditions were different, the average predicted EME was similar and the effects of herd, parity, lactation stage and milk yield on EME traits were following the expected patterns according to nutritional and physiological knowledge. We also found all of these EME traits to have a genetic background, with a heritability of 0.12 to 0.25 according to the different traits [18], as did other researchers using different methods (0.17 to 0.25 according to the different traits, as summarized by Brito et al. [19]). The milk FA-based EME predictions seem to have correlation coefficients with the respiratory chamber gold standard values in the same range of the methods analyzing methane at farm level. On the other side, this indirect method is based on a causative relation between the milk FA profile and the rumen fermentation pattern. However, the need for gas chromatography analysis of milk samples remains an obstacle to including EME traits in dairy cattle selection programs because of the cost and manpower required.

The wide availability of milk Fourier-transform infrared (FTIR) spectra obtained from samples collected for milk recording schemes motivated researchers to use these to devise proper calibrations for EME traits. However, controversial results were reported [20,21,22] when milk FTIR spectra were used for prediction of EME traits. In all these cases, the EME reference values were not obtained with the gold standard respiratory chamber methods, but from analyses of air sampled near the nostrils of cows in the practice conditions. On the other side, data obtained in respiratory chambers, even though precise, referred to a small number of cows and diets and rearing conditions very different from commercial milk yield to yield robust FTIR calibrations and even weaker validations. A large effort for using respiration chambers reference data, developing milk FTIR calibrations and predicting daily methane production, combined the results of 148 cows from research facilities of five European countries and, including in the equations also the constant, linear and quadratic Legendre polynomials of days in milk, an R^2^ of calibration of 0.64 was obtained [23].

Taking a different approach, we used FA-derived EME estimates as reference values and obtained FTIR calibrations with validation accuracies [24] similar to those of the original equations from respiration chamber data (R^2^ 0.47 and 0.54) [16]. In addition to the FTIR calibrations directly predicting the five EME traits investigated (direct predictions), we also tested eight indirect methods based on FTIR predictions of milk FAs, percentage cheese yield and daily milk yield (dMY, kg/d) with variable results. It is clear that the number, representativeness, and accuracy of reference data remains the major unsolved problem of any FTIR based EME prediction.

To give a further contribution to the knowledge of the complex relationships between rumen fermentation, milk composition and absorbance properties and environmental impact of dairy cows, in the present study, we use the information obtained from the FTIR calibrations and genetic estimates of reference EME traits obtained from gas-chromatographic analysis of milk FA profile with the aims of: (i) estimating the heritability of five EME traits obtained directly from milk FTIR spectra, and of eight EME traits obtained indirectly from milk FTIR spectra; (ii) estimating the additive genetic, phenotypic, herd and residual correlations between the five GC FA (fatty acids obtained by gas-chromatography)-based reference and the 13 directly and indirectly FTIR-predicted EME traits; and (iii) assessing their possible use for selective and non-selective purposes.

## 2. Materials and Methods

### 2.1. Animals, Samples and Analyses

Details of the animals and farms can be found in our previous study [17]. Briefly, we carried out the study using milk samples taken during the evening milking of 1158 Brown Swiss cows, across different parities and lactation stages, reared in 85 herds (15 cows per herd, with few exceptions) located in Trento Province (northeastern Italian Alps), representing different dairy farming systems, from traditional to modern ones.

Detailed FA profiles of all the milk samples were obtained by the gas-chromatographic (GC) method described in detail in previous studies [25,26,27]. Briefly, FA methyl esters were prepared using the direct extraction and alkali-catalyzed trans-methylation procedure. FA composition was determined using a ThermoQuest gas chromatograph (Thermo Electron Corp., Waltham, MA, USA) fitted with a flame-ionization detector and a high polar fused-silica capillary column (Chrompack CP-Sil88 Varian, Middelburg, the Netherlands; 100 m, 0.25 mm i.d.; film thickness 0.20 μm). Inter- and intra-assay coefficients of variation were also calculated using a butter reference standard (BCR 164; Commission of the European Communities, Community Bureau of Reference, Brussels, Belgium) with the analytical limit of detection set at 0.001% above that of the total amount of FAs. Milk FA composition was expressed in grams per 100 g of total FAs. In conformity with van Lingen et al.’s [16] equations, we examined the following FAs: 4:0 (butyric acid), 16:0iso (iso-palmitic acid), 18:1*t*10 (iso-oleic acid), 18:1*t*11 (vaccenic acid), 18:1*c*9 (oleic acid), and 18:2*c*9,*c*12 (linoleic acid).

### 2.2. Definition of EME Phenotypes Used as Reference

In accordance with Bittante et al. [17], the EME traits used here as reference values to build the models of prediction from FTIR spectra (GC-Reference) were derived from GC measured FA profile as follows:

- Methane yield: EME (g) per kg DMI (CH_4_/DMI_FAGC_), predicted from the FAs obtained by gas chromatography according to van Lingen et al.’s [16] equation:
CH_4_/DM_FAGC_ (g/kg) = 23.39 + 9.74 × C16:0*iso* − 1.06 × C18:1*trans*-10 + *trans*-11 − 1.75 × C18:2*cis*-9,*cis*12
  where C16:0*iso* is iso-palmitic acid, C18:*trans*-10 + *trans*-11 is the sum of the iso-oleic and vaccenic acids, and 18:2*cis*-9,*cis*-12 is the linoleic acid of milk, all expressed as % of the sum of all milk FAs.
- Methane intensity-milk: EME (g) per kg CM (CH_4_/CM_FAGC_), predicted according to van Lingen et al.’s [16] equation:
CH_4_/CM_FAGC_ (g/kg) = 21.13 − 1.38 × C4:0 + 8.53 × C16:0*iso* − 0.22 × C18:1*cis*-9 − 0.59 × C18:1*trans*-10 + *trans*-11
  where C4:0 is butyric acid, and C18:1*cis*-9 is oleic acid, all expressed as % of the sum of all milk FAs.
- Methane production: daily EME (g) per cow (dCH_4_), calculated as:
dCH_4_ (g/d) = CH_4_/CM_FAGC_ × dCMY
- Methane intensity-cheese: EME (g) per kg of fresh cheese (CH_4_/CY_CURD_), calculated as:
CH_4_/CY_CURD_ (g/kg) = dCH_4_/dCY_CURD_
  where dCY_CURD_ is the daily yield of cheese per cow (kg/d).
- Methane intensity-cheese solids: EME (g) per kg of cheese solids (CH_4_/CY_SOLIDS_), calculated as:
CH_4_/CY_SOLIDS_ (g/kg) = dCH_4_/dCY_SOLIDS_
  where dCY_SOLIDS_ is the daily yield of cheese dry matter per cow (kg/d).

### 2.3. Infrared Spectra Acquisition and Editing

Details of the milk spectra acquisition, editing, calibration and validation procedures were described in our previous study [24]. Briefly, within 20 h of milking, the full FTIR spectrum of absorbance was retrieved from an FTIR spectrometer (FT 6000; Foss Electric, Hillerød, Denmark), and 1060 absorbance values were recorded for each milk sample, covering the infrared wave numbers ranging from 5000 × cm^−1^ (corresponding to a wavelength of 2.0 μm) in the near-infrared (NIR) or short-wavelength infrared (SWIR) subdivision of the infrared region, through the mid-infrared (MIR) region, to wave number 930 × cm^−1^ (corresponding to a wavelength of 10.8 μm) in the long-wavelength infrared (LWIR) subdivision [28]. After centering and standardization, the spectra were not subjected to any further mathematical pretreatment.

### 2.4. Prediction of EME Traits Directly from FTIR Milk Spectra

The simplest way of predicting EME traits from FTIR milk spectra is to consider the GC reference EME traits as the dependent variables to be regressed on the FTIR absorbance spectra, considered as independent variables. As each of the 5 GC-reference EME traits is obtained as the result of equations combining different sources of information (4 quality traits, 4 daily yield traits and 6 milk FA proportions), this approach tries to predict directly the product of equations, losing the details, specificities and accuracies of each term of the equation. Separate models were fitted for the 5 EME traits considered here. We used a Bayesian model (BayesB model) implemented in the BGLR package of the R software, as previously described by Ferragina et al. [29]. Phenotypes were regressed on standardized spectra covariates using the linear model:

$$y_{i}=\beta_{0}+\sum_{j=1}^{1060}x_{\mathrm{ij}}\beta_{j}+\varepsilon_{i}$$

where $\beta_{0}$ is an intercept, $\left\{x_{\mathrm{ij}}\right\}$ are standardized FTIR spectra-derived wavelength data (j=1,…,1060), $\beta_{j}$ are the effects of each of the wavelengths and $\varepsilon_{i}$ are model residuals assumed to be *iid* (independent and identically distributed) with normal distribution centered at zero with variance $\sigma_{\varepsilon }^{2}$. The accuracy of the model and the prediction equation were assessed with a 10-fold training-testing procedure [24].

The above-described Bayesian approach was used for direct (Direct IR) prediction of the 5 EME traits, where “Direct” refers to the use of the 5 GC-Reference EME data as dependent variables regressed against the 1060 absorbance values of each FTIR spectrum (R^2^_CV_: coefficient of determination of cross-validation; RMSE_CV_: root mean square error of cross-validation [24]):

- Predicted methane yield (CH_4_/DMI_IR_, g/kg; R^2^_CV_: 0.49; RMSE_CV_: 1.18);
- Predicted methane intensity-milk (CH_4_/CM_IR_, g/kg; R^2^_CV_: 0.57; RMSE_CV_: 1.17);
- Predicted methane intensity-fresh cheese (CH_4_/CY_CURD-IR_, g/kg; R^2^_CV_: 0.55; RMSE_CV_: 11.2);
- Predicted methane intensity-cheese solids (CH_4_/CY_SOLID-IR_, g/kg; R^2^_CV_:0.47; RMSE_CV_: 22.6);
- Predicted daily methane production (dCH_4-IR_, g/d; R^2^_CV_: 0.36; RMSE_CV_: 86.0).

### 2.5. Prediction of EME Traits Indirectly Based On FTIR Prediction of Other Milk Traits

An alternative to the previously described direct IR (infrared) predictions is not to predict from milk spectra directly the products of GC reference equations, but to predict each term of equations and then to obtain the product of equations based on IR-predicted terms. The same IR model previously described for direct IR predictions was also applied to predict the 14 informative traits used for calculating the reference EME traits (4 quality traits, 4 daily yield traits and 6 milk FA proportions) [24]. The reference milk fat and protein contents shown in Table 1 were obtained by the spectrometer through the calibration equations pre-installed by the supplier company according to ICAR (International Committee for Animal Recording) official methods [30], whereas the cheese yields, expressed as the weight of fresh cheese (%CY_CURD_) or cheese solids (%CY_SOLIDS_) as percentages of the weight of the milk processed, were obtained as previously described by Cecchinato et al. [31]. The dCY were obtained by multiplying dMY by the corresponding %CY.

The IR predictions of informative traits were used to develop 8 “indirect” procedures for EME prediction (r; Pearson correlations with the corresponding reference values [24]):

- CH_4_/DMI_FAIR_ (r: 0.78), using van Lingen et al.’s [15] same equations as for calculating the GC reference data, but with FA_IR_ instead of FA_GC_;
- CH_4_/CM_FAIR_ (r: 0.82), again using van Lingen et al.’s [15] equations, but with FA_IR_ instead of FA_GC_;
- CH_4_/CY_CURD-IR-IR_ (r: 0.67), using the same procedure as for the reference values, but substituting the reference CH_4_/CM_FAGC_ with its direct IR prediction (CH_4_/CM_IR_), and the measured %CY_CURD_ with its IR prediction;
- CH_4_/CY_SOLIDS-IR-IR_ (r: 0.62), using the same procedure as for the reference values, but substituting the reference CH_4_/CM_FAGC_ with its direct IR prediction (CH_4_/CM_IR_), and the measured %CY_SOLIDS_ with its IR prediction;
- dCH_4-CM-IR_ (r: 0.96), by multiplying the measured corrected milk yield (dCMY) by the direct-IR methane intensity-milk (CH_4_/CM_IR_);
- dCH_4-CM-FAIR_ (r: 0.96), by multiplying the dCMY by the indirect-IR methane intensity-milk based on FA_IR_ (CH_4_/CM_FAIR_);
- dCH_4-IR-IR_ (r: 0.65), by multiplying the IR-predicted daily corrected milk yield (dCM_IR_) by the CH_4_/CM_IR_;
- dCH_4-IR-FAIR_ (r: 0.63), by multiplying the dCM_IR_ by the CH_4_/CM_FAIR_.

### 2.6. Databases for Genetic Analyses

Two databases were created for the genetic analyses: the first with the phenotypes, the second with pedigree information.

An important issue was to avoid possible inflation of the genetic parameters as a result of employing, for genetic analyses, the infrared predictions used to develop the calibration equations. Every prediction used for equation calibration (training subsets) was excluded from genetic analyses. The database was created using only the independent predicted phenotypes used for validation of equations (testing sub-sets). As the training–testing procedure used 80% of the samples for training and 20% for testing and was repeated 10 times randomly for each trait, about 10% of the milk samples were never used in the testing procedure and were not included in the database of phenotypes for genetic analyses, whereas in the case of samples used more than once for testing, the averages of these phenotypes were included.

Data concerning the cows and herds were provided by the Superbrown Consortium of Bolzano and Trento (Italy), and pedigree information was supplied by the Italian Brown Swiss Cattle Breeders Association (ANARB, Verona, Italy). We included cows with phenotypic records available for the investigated traits and all known ancestors. Each sampled cow had at least 4 generations of known ancestors, and the pedigree file included 8845 animals. The number of sires was 1326; of these, 264 had progeny with records in the data set (each sire having between 2 and 80 daughters).

### 2.7. Statistical Analysis

The statistical models used for estimating the genetic parameters of directly and indirectly FTIR-predicted EME traits was described in detail in our previous study on the genetics of reference EME traits [18]. For all traits, the model accounted for the non-genetic effects of herd/date (85 levels), days in milk (Days in milk, DIM: class 1, <60 d; class 2, 60 to 120 d; class 3, 121 to 180 d; class 4, 181 to 240 d; class 5, 241 to 300 d; class 6, >300 d), and parity (1 to 4 or more).

The genetic determinism of the EME traits and their predictors (y) was investigated by analyzing the data with the following hierarchical model:

(1) $$y=Xb+Z_{1}h+Z_{2}a+e$$

where y is the vector of phenotypic records with dimension *n*; X, $Z_{1}$ and $Z_{2}$ are the appropriate incidence matrices for systematic effects (b), herd/date effects (h), and polygenic additive genetic effects (a), respectively. The priors for b and the variance components were assumed to be flat.

A standard Bayesian approach was used to analyze all the models [18].

We estimated the phenotypic, additive genetic, herd/date and residual correlations between the directly or indirectly predicted EME traits and the corresponding reference values by conducting a set of bivariate analyses that implemented model [1] in its multivariate version.

In this case, the traits involved were assumed to jointly follow a multivariate normal distribution, along with the additive genetic, herd/date and residual effects. The corresponding prior distributions for these effects were:

$$a|G_{0},\;A\;\sim \;MVN\left(0,G_{0},⨂A\right)$$

$$h|H_{0},\;\sim \;N\left(0,H_{0},⨂I_{n}\right)$$

$$e|R_{0},\;\sim \;N\left(0,R_{0},⨂I_{m}\right)$$

where $G_{0},\;H_{0}\;,\;R_{0}$ are the corresponding variance-covariance matrices between the traits involved and a, h and e are vectors of dimension equal to the number of animals in the pedigree (*n* and *m*) times the number of traits considered.

Marginal posterior distributions of all unknowns were estimated using the Gibbs sampling algorithm. The TM program (http://snp.toulouse.inra.fr/~alegarra) was used for all Gibbs sampling procedures. The chain lengths and burn-in period were assessed by visual inspection of the trace plots. After some preliminary analyses, chains of 850,000 samples were kept, and the burn-in period was set at 50,000, after which every 100th sample was retained.

The posterior mean was used as the point estimate for all parameters. The lower and upper bounds of the highest 95% probability density regions (HPD95%) were obtained from the estimated marginal densities of heritability estimates. For the phenotypic, genetic, herd and residual correlations, in addition to calculating the mean of each marginal posterior distribution, we also estimated the probability of each mean being greater than 0 when the mean was positive, or lower than 0 when the mean was negative (*p*). We considered all estimates with *p* greater than 95% as “relevant” correlations.

Intra-herd heritability ($h^{2}$) was computed as:

$$h^{2}=\frac{\sigma_{a}^{2}}{\sigma_{a}^{2}+\sigma_{e}^{2}}$$

The proportion of the total variance due to herd/date (h_herd_) was computed as:

$$h_{herd}=\frac{\sigma_{h}^{2}}{\sigma_{a}^{2}+\sigma_{h}^{2}+\sigma_{e}^{2}}$$

where $\sigma_{a}^{2}$, $\sigma_{h}^{2}$ and $\sigma_{e}^{2}$ are the additive genetic, herd/date and residual variances, respectively.

We also computed the phenotypic (r_P_), additive genetic (r_G_), herd/date (r_H_) and residual (r_E_) correlations between a given predicted trait and its corresponding reference trait.

## 3. Results

### 3.1. Variance Components, and Estimates of Heritability and Herd Incidence

Point estimates of the marginal posterior densities for the additive genetic, herd/date and residual variances were lower in the case of the direct IR and indirect IR predictions than the GC reference values for both methane yield and methane intensity—milk (Table 2). In the case of methane intensity—fresh cheese, methane intensity—cheese solids (Table 3), and daily methane production (Table 4), there was greater variation in the variances according to each trait.

The intra-herd heritabilities ($h^{2}$) of the traits also varied according to the proportions of the variance components in the different cases. For methane yield and intensity—milk, the direct IR predictions were slightly less heritable than the GC reference phenotypes, whereas the indirect FAIR predictions were more heritable. About 10 percentage points separated the heritabilities of direct IR and of indirect FAIR (fatty acids obtained by infrared spectroscopy) predictions, in favor of the latter, for both EME traits.

We obtained different results for methane intensity—fresh cheese and methane intensity—cheese solids (Table 3). In the case of the former EME trait, heritability was greater for the infrared predictions than for the GC reference values, whereas the reverse was found for CH_4_/CY_SOLIDS_. For both traits, better results were obtained with the direct IR methods than with the indirect IR method.

In the case of daily methane production per cow, the heritability estimates of all the infrared-based predictions were greater than the GC reference phenotype (Table 4). The variability in heritability was greater for the predicted traits that the GC reference traits when expressed as the standard deviation, but smaller in terms of the variability coefficient.

The herd/date variance component was always very large and often similar to or greater than the residual variance, so that the contribution of the herd/date effect to total phenotypic variance varied from 37% to 71%. Methane yield (Table 2) was the most affected by herd/date, followed by the methane intensities (Table 2 and Table 3), and then by methane production (Table 4). The herd/date effects on the direct and indirect EME trait predictions were similar to those on their corresponding GC reference traits, with the exception of the indirect IR, IR predictions of methane intensity—fresh cheese and methane intensity—cheese solids, which exhibited lower herd/date effects than in the case of the direct IR and reference predictions due to their larger residual variances.

### 3.2. Phenotypic, Additive Genetic, Herd and Residual Correlations

Table 2, Table 3 and Table 4 show the point estimates of the marginal posterior densities of the phenotypic, additive genetic, herd and residual correlations between the infrared-predicted EME traits and their corresponding reference values. The two indirect predictions of methane production obtained by multiplying the measured daily milk yield (dCMY) by the direct IR- or indirect FAIR-predicted methane intensity—milk presented very high (0.91 to 0.98) phenotypic, genetic, herd/date and residual correlations with their corresponding reference methane production values, obtained by multiplying the dCMY by the GC reference methane intensity-milk. This is because the measured dCMY is present in both EME traits and is characterized by a much larger variability coefficient respect to the two methane intensity—milk traits. All the other predictions obtained directly and indirectly from milk FTIR spectra presented moderate–high (0.52 to 0.75) phenotypic correlations with their corresponding reference values (Table 2, Table 3 and Table 4), more variable (0.22 to 0.93) genetic correlations, high (0.82 to 0.94) herd/date correlations, and low–moderate (0.16 to 0.39) residual correlations. The high variability in the genetic correlations was due to the fact that, unlike the two methane production values obtained from the measured trait, the two values obtained from IR-predicted daily milk yield had very low (0.22–0.29) genetic correlations with the reference values (Table 4).

## 4. Discussion

The importance of different EME traits for the genetic improvement of dairy cattle to mitigate climate change depends on their overall effect and on the cost/revenue ratio. On the one hand, a simple, rapid, precise and inexpensive method of prediction at the population level needs to be available, while on the other hand, the relationships with the overall selection goals of the population in question must be taken into account. From the results of nine experiments carried out in respiration chambers, van Gastelen et al. [32] concluded that calibrations for prediction of EME traits based on milk fatty acid profile have a greater potential (+0.01 to +0.07 in the R2 according to the predicted trait) than those based on milk FTIR spectra. They also concluded that both models are robust but that additional measurements are needed for improving accuracy and robustness of both methods. Infrared prediction, on the other side, is the simplest, fastest and least expensive among the available methods for indirectly predicting the traits of concern to the dairy cattle industry. These predicted traits could be used at population level at very low additional cost, as they could be provided for the animals registered in milk recording schemes for which the composition of milk is routinely analyzed by FTIR spectrometry [31]. The heritability estimates of the predictions of EME traits obtained in this work fall within the range of the 36 different estimates reported in 10 studies on dairy cows that were the subject of a meta-analysis proposed by Brito et al. [19].

As with every other type of indirect prediction, the effectiveness of infrared predictions should be evaluated on the basis of their genetic parameters. Comparing the heritability of predicted traits with their corresponding reference traits is clearly important, but it should be borne in mind that they are affected by the number of observations used to establish the calibration equations and to validate them [33]. Moreover, they depend on the repeatability and reproducibility of the gold standard analysis employed.

Heritability is not, on its own, a sound evaluation of the effectiveness of substituting the reference trait with the predicted trait for genetic selection as it does not provide any information on the relationship between the reference and the predicted trait [34]. It is only with the genetic correlations between the measured and predicted values that a clear picture of the traits of concern emerges. The major limit of EME trait predictions is the unsolved problem of the reference method. In fact, the gold standard method based on respiration chambers, as previously said, is precise, but can generally be applied on few cows and few diets, and the chamber conditions are very different from that of commercial farms. As seen, the prediction methods that can be used at population level for measuring the ratio between methane and carbon dioxide near the head of cows are much less precise and present correlations with the gold standard not much different from those characterizing the indirect prediction method based on milk FA profile. This last method is characterized by strong causal relationships with rumen fermentations and the ability to capture and quantify the expected environmental effects of a cow’s feeding, parity and lactation stage [17]. In any case, the results obtained need to be treated with great prudence and comparisons with other reference data should be made.

### 4.1. Infrared Prediction of Methane Yield per Unit of Dry Matter Consumed

The methane yield estimated from this dataset using the GC reference method ($h^{2}$ = 0.274; Table 2) showed that this EME trait had the highest intra-herd heritability, which was slightly higher than the average value ($h^{2}$ = 0.23 ± 0.03), summarized by Brito et al. [19]. However, the usefulness of the infrared prediction of this trait is questionable because, even though the heritability is high, the genetic correlation with the values obtained using the reference method is only moderate (0.45–0.60; Table 2). In any case, if GC FA profile is not available, indirect IR prediction of the proportions of informative FAs in the milk fat and the use of van Lingen et al.’s [16] equation seems preferable to direct IR prediction of methane yield from milk FTIR spectra as the heritability is higher and the genetic correlation with the GC reference trait is stronger.

In terms of its application in the dairy chain, methane yield seems to be more useful for non-genetic purposes, e.g., for evaluating the ecological footprints of different feeding regimes within different dairy systems and farms (r_H_ 0.92–0.93, Table 2). It worth noting that the dairy system, and specifically feeding regime, affects the phenotypic expression of all EME traits [17], but it influences the heritability value only of CH_4_/DMI [18]. This is further confirmed by the very high herd/date effect exhibited by this trait, which shows it to be highly dependent on the environment, management and feeding practices of individual farms, and by the high herd/date correlation between the prediction and reference methods. Moreover, its usefulness for the genetic improvement of dairy cows is hampered by the lack of daily feed intake records for individual animals at the field level, precluding estimation of actual daily enteric GHG emissions, and because of poor knowledge of the relationship between this trait and feed intake and efficiency of utilization at the individual animal level.

### 4.2. Infrared Prediction of Methane Intensity per Unit of Milk or Cheese Produced

With respect to the contribution made by EME to the dairy industry’s overall environmental impact, reducing methane intensity per unit of dairy product may be considered the ultimate goal. The main determinant of the dairy chain’s size and impact is the market’s demand for fluid milk and/or other dairy products, whereas, given the market’s needs, the number of cows and farms needed depends on their respective levels of productivity and size.

In this study, we found the heritability of methane intensity—milk to be slightly lower than that of methane yield, and almost identical to the average value reported by Brito et al. [19] in their meta-analysis. On average, infrared predictions of this trait have similar heritabilities and favorable genetic correlations with the values obtained using the GC reference method. Again, the Indirect FA_IR_ method returns a greater heritability than the direct IR method, but the latter’s lower value is compensated for by a greater genetic correlation with the reference values, so that no one method has a clear advantage over the other.

A very preliminary trial investigating the potential of predicting methane intensity—milk using milk FTIR spectra was carried out by Dehareng et al. [20] using a different reference method. Instead of using a respiration chamber, they released an SF_6_ tracer into the rumen then analyzed the air between the nostrils and the noses of 11 cows. The results were encouraging, so the authors increased the number of cows to 142 and made 446 air analyses [21]. The resulting heritability coefficient of methane intensity (after logarithmic transformation) estimated at the population level was very similar, on average, to our direct IR predictions; they also found that heritability increased over the course of lactation [35]. Denninger et al. [36] found that cows categorized as high and low emitters on the basis of FTIR predictions continue to maintain emission differences between the two groups with time, both in terms of FTIR predicted and of GreenFeed measured EME values.

For countries like Italy and some other European countries, where the dairy chain is predominantly oriented to cheese production rather than fluid milk, the final goal should be to reduce methane intensity-cheese, leaving aside any differences in the cheese-making efficiencies of milk from different dairy systems, farms, breeds and individual animals within breed [37]. When based on the GC reference method, the heritability of methane intensity—cheese, whether fresh cheese or cheese solids, is lower than that of fluid milk (Table 2 and Table 3). It should be noted that, with respect to IR predictions, direct IR prediction of methane intensity-fresh cheese has a favorable heritability (0.267) and good genetic correlations (0.93) with the reference values. This method is, therefore, probably preferable to the indirect IR-IR method with respect to this trait, and to both the methods for predicting methane intensity-cheese solids (Table 3).

### 4.3. Infrared Prediction of Daily Methane Production per Cow

The daily methane production of individual cows may seem to be the most suitable EME trait for the genetic improvement of dairy populations, and indeed it has often been proposed as the main selection goal for mitigating climate change [38]. There are, however, two major obstacles to using this trait as a selection goal: one theoretical, and one practical.

The first obstacle to the use of daily methane production in selective breeding is the positive genetic correlation with daily milk and cheese yields [18,39,40]. This obviously means that including this trait, with a negative sign, in a selection index together with daily milk/fat/protein yields would reduce the efficiency of the selection and slow down the genetic improvement of milk-to-cheese traits. In a large survey on commercial dairy farms cows with low methane production, as identified through milk FTIR spectra, daily methane production was showed to be associated with lower milk yield, lower milk quality, suboptimal reproduction and health performance, higher culling rate and lower economic gross margin per cow and per liter milk [41]. Methane intensity, on the other hand, exhibited much smaller, non–relevant correlations with milk production [38,42,43].

The second obstacle is the fact that a dairy cow’s daily methane production depends, first of all, on the quantity (and quality) of the feed ingested, which in turn depends, in particular, on the cow’s size and production level [44]. Trying to directly predict the daily methane emission of a cow using the FTIR spectrum of a sample of the milk it produces and without any other information is, in the first place, like trying to estimate a cow’s size, production level and feed intake from the characteristics of its milk. This explains the modest validation performance of the direct calibration of methane production from FTIR spectra [24]. The fact that the heritability of predicted methane production is greater than that of the reference values could be interpreted as due to the good repeatability of infrared milk spectra [34], and to the fact that this heritability could have more to do with that of the infrared absorbance spectrum in itself [28] than that of the reference trait. This interpretation finds further support in the modest genetic, but also phenotypic, correlations between the predicted and GC reference values.

An approach similar to that taken in this study was previously used by Kandel et al. [45], who adopted the four prediction equations proposed by Chilliard et al. [46], and was probably the first study to look at the possibility of predicting EME traits from the milk fatty acid profile for genetic purposes. These equations, which were not obtained in a respiration chamber, were derived from a single experiment on cows fed the same diet, which included different linseed products. Kandel et al. [45] used only the equations based on FAs de novo synthesized in the udder (saturated linear chain even FAs, 6:0 to 16:0) because they are very easily predicted by FTIR, but it is now known that these FAs tend to overestimate CH_4_ production and are not easily applicable to different conditions [13,47]. In any case, their direct IR predictions of methane production had moderate–medium heritability values, but the genetic correlations between the reference and predicted values were not reported, so the results should be treated with caution.

As expected, the situation in our study did not improve when we substituted the direct FTIR prediction of methane production with contemporary predictions of milk yield and methane intensity—milk, and their values multiplied by each other (Table 4). As we have seen, the situation changes completely when measured milk yield is multiplied by predicted methane intensity—milk (Table 4): the heritabilities of these indirect predictions are still greater than those of the GC reference values, but their genetic (and phenotypic, and herd/date and residual) correlations are close to unity. This has to do with the fact that milk yield has a much greater variability than CH_4_/CM_IR_, and it was used both in GC reference and IR-predicted traits. Similar good estimates of daily methane production have been obtained by Engelke et al. [48] using FTIR predicted fatty acids and daily corrected milk yield. It makes no practical sense to try to predict a cow’s milk production from an infrared spectrum of its milk when the measured yield is available.

It worth noting that Vanlierde et al. [21] improved (R^2^_CV_ = 0.70) the preliminary FTIR prediction of methane production obtained by Dehareng et al. [20] by increasing the number of cows sampled, and including a third order DIM function in the prediction equation, thereby indirectly taking into account the variation in daily milk yield; the heritability coefficient of methane production they found with this method was intermediate between our direct IR predictions and those obtained by multiplying dMY by methane intensity-milk [35]. This genetic parameter was found to be relatively constant during lactation.

### 4.4. Use of FTIR Predictions of EME Traits in Genetic Improvement of Dairy Cattle

As seen, the gold standard EME phenotypes from respiration chambers cannot be obtained on a large number of cows and in field conditions. The predictions of gold standard EME traits obtainable on large numbers of cows in conditions similar to the practice are based on two different approaches: the analysis of the ratio between CH_4_ and CO_2_ of the air sampled (for few minutes, a few times per day) near the nostrils of the cows (“sniffers” in automatic feeding or milking stations, portable laser detectors, portable infrared gas detectors, etc.), or the analysis of the fatty acid profile of milk as an indicator of rumen fermentations [3,12]. None of these methods actually measures EME produced by cows because they do not measure the quantity of gas daily emitted by cows, but they are calibrated, with variable accuracy, on gold standard data obtained in respiration chambers [12,13]. Even though these methods are feasible at farm level, they require expensive instrumentations (air analyzers) or milk sampling and laborious analyses (fatty acid profile). The predictions based on FTIR spectra of milk samples, on the contrary, could be obtainable on all samples analyzed during milk recording on the entire recorded dairy cattle population at every test date with the only additional cost the calibration equations and their periodic maintenance. The FTIR predictions based on gold standard respiration chambers data [23] are based on a limited number of precise phenotypes recorded in an environment far from the practice. On the other hand, the predictions based on predictions obtained on commercial farms, like those used in the present study or obtained from air analyses, could rely on much more numerous and cheap reference values, but are predictions of predictions. The EME predictions (based air analysis or milk fatty acid profiles) are often scarcely correlated among them [8] or can yield biased results [49], whereas the FTIR predictions based on different reference methods are not yet compared. The improvement of accuracy of FTIR predictions of EME traits, their validation with respiration chambers data and their comparison represent important fields for future research.

In any case, the rapid availability of a very large number of EME predictions with negligible costs represents an opportunity that dairy sector cannot loose, also because of the ethical commitment toward society and environment. Even though their accuracy could be modest, their heritability and genetic correlations with reference data, as found also in this study, seem to indicate their effective possible contribution to the mitigation of global climate change. The last question, representing a limitation to their inclusion in selection indice’s is the knowledge of the genetic correlations between EME traits and the other traits included in selection indices (or better, objective of selection).

In previous research on the genetics of EME predictions based on milk fatty acid profile [18], we concluded that daily milk and cheese yield traits were all, as expected, highly positively (unfavorably) correlated with estimated daily methane production from the phenotypic, genetic, herd and residual point of view. In contrast, milk production and quality traits were negatively (favorably) correlated with the estimated methane yield and intensities [18]. In this study on milk FTIR spectra-based predictions, we estimated genetic correlations with production traits that showed patterns similar to those based on milk fatty acid profile (data not shown). The overall economic impact of inclusion of EME traits on selection indices is dependent on which EME trait is included in the selection program.

In particular, on the base of this study, it appears that methane yield per kg of dry matter ingested by the cows can be easily predicted, and the best results are not obtained through direct prediction from milk FTIR spectra, but rather from an indirect method based on the prediction of some informative FAs in milk fat, i.e., a prediction from a prediction. In any case, this EME trait seems to have greater value for non-genetic purposes, especially for evaluating feeding practices.

Reducing methane intensity per unit of milk produced should be the major objective of genetic mitigation of the contribution made by cows to climate change at the level of the overall dairy chain, provided that fluid milk is the main product. In this case, both direct FTIR prediction and indirect prediction through informative FAs should be considered as they are highly correlated with the predictions based from milk fatty acid profile. If the dairy chain operates mainly within a market requiring large amounts of cheese, methane intensity could be directly predicted per kg of fresh cheese produced with good prospects. The inclusion of these EME traits in selection indices is favored also by the small but favorable correlations with cow’s productive traits [18].

Lastly, daily methane production per cow, although seemingly attractive, does not appear to be a feasible objective of selection due to positive (unfavorable) correlations with milk production [39] and low genetic correlations between the measured and predicted values [18]. However, if this EME trait has to be obtained, a much better option appears to be multiplying the measured milk yield by the predicted methane intensity—milk, instead of predicting directly the daily methane production.

It is worth noting that the inclusion of FTIR-based predictions in selection programs is favored also by the fact that the new phenotype could be simply treated as a new additional trait in the databases of production and quality traits obtained during milk recording operations [50]. Therefore, it would be very easy to estimate the breeding values at population levels with few modifications of the statistical procedures normally used. The contribution of the genome-wide approach to selection should be better evaluated [51], but it seems rather simple from the technical point of view.

## 5. Conclusions

In conclusion, within all the limits of this field of research, from this study, it appears that FTIR of milk spectra could yield predictions that are heritable and genetically correlated with EME traits estimated on the basis of milk fatty acid profile. This, coupled with the high throughput, rapidity, low cost and ease of use of the novel phenotypes, makes FTIR predictions candidates to be rapidly included in the selection programs of dairy cows’ populations. Further research on large populations at the field level and experimental validation with other direct EME prediction methods, and particularly with respiration chamber balances, is required to test these preliminary results in different conditions and with different breeds.

## Figures and Tables

**Table 1:** Descriptive statistics for the milk quality and daily yield traits.

Item	Mean	SD	CV, % ^1^
*Quality traits, %* ^2^			
Milk fat	4.22	0.70	16
Milk protein	3.70	0.43	12
%CY_CURD_	14.96	1.89	13
%CY_SOLIDS_	7.21	0.93	13
*Daily yield traits, kg/d* ^3^			
dMY	24.8	8.0	32
dCMY	25.8	8.2	32
dCY_CURD_	3.69	1.17	31
dCY_SOLIDS_	1.76	0.58	33

^1^ CV = coefficient of variation; ^2^ %CY_CURD_ = wt of fresh cheese as % of processed milk; %CY_SOLIDS_ = wt of cheese solids as % of processed milk; ^3^ Daily yield traits: dMY = daily milk yield; dCMY = daily fat and protein corrected milk yield, obtained multiplying dMY by a correcting factor (CF) where CF=0.337 + 0.116 × milk fat (%) + 0.06 × milk protein (%); dCY_CURD_ = daily production of fresh cheese per cow; dCY_SOLIDS_ = daily production of cheese solids.

**Table 2:** Estimates of additive genetic ($\sigma_{a}^{2}$), herd/date ($\sigma_{h}^{2}$), and residual ($\sigma_{e}^{2}$) variances, heritability, and herd/date effect of GC reference values based on GC FA profile, and of direct IR and indirect IR predictions of methane yield and methane intensity milk based on milk FTIR spectra, and additive genetic (r_G_), herd (r_H_), residual (r_E_), and phenotypic (r_P_) correlations between GC reference and IR predicted methane emission traits ^1^.

Item	Methane Yield			Methane Intensity Milk		
	GC Reference CH_4_/DMI	Direct IR CH_4_/DMI_IR_	Indirect IR CH_4_/DMIFA_IR_	GC Reference CH_4_CM	Direct IR CH_4_CM_IR_	Indirect IR CH_4_CMFA_IR_
Cows, N	958	958	958	958	958	958
Descriptive statistics:						
Mean	21.30	21.34	21.34	14.18	14.19	14.18
SD	1.61	1.23	1.13	1.77	1.46	1.33
Variances:						
$\sigma_{a}^{2}$	0.204	0.114	0.135	0.228	0.122	0.162
$\sigma_{h}^{2}$	1.819	0.952	0.859	1.486	0.902	0.826
$\sigma_{e}^{2}$	0.539	0.367	0.250	0.963	0.642	0.443
Heritability:						
Mean ^2^	0.274	0.237	0.351	0.191	0.160	0.267
HPD95 ^3^	0.08; 0.46	0.05; 0.42	0.11; 0.58	0.002; 0.37	0.01; 0.32	0.07; 0.46
Herd/date effect:						
Mean ^2^	0.706	0.661	0.687	0.551	0.537	0.574
HPD95 ^3^	0.63; 0.77	0.58; 0.73	0.61; 0.75	0.46; 0.63	0.44; 0.62	0.48; 0.66
Correlations ^4^ with GC-Reference:						
r_P_	-	**0.73**	**0.73**	-	**0.72**	**0.72**
r_G_	-	**0.45**	**0.60**	-	**0.88**	**0.73**
r_H_	-	**0.93**	**0.92**	-	**0.92**	**0.93**
r_E_	-	**0.22**	**0.16**	-	**0.38**	**0.38**

^1^ Heritability defined as intra-herd/date heritability $h^{2}=\;\sigma_{a}^{2}/(\sigma_{a}^{2}+\sigma_{e}^{2})$; herd/date effect defined as${\;h}_{\mathrm{herd}}={\;\sigma }_{h}^{2}/(\sigma_{a}^{2}+\sigma_{h}^{2}+\sigma_{e}^{2})$; Direct FTIR predictions are based on specific calibrations of FTIR milk spectra, whereas indirect ones are based on FTIR predictions of the concentration in milk of 6 informative fatty acids used to estimate methane emission traits through equations from a meta-analysis of data from dairy cows in respiration chambers [17]; ^2^ Mean = mean of the marginal posterior distribution; ^3^ HPD95 = bounds of the 95% high posterior density interval; ^4^ Boldface indicates correlations with >95% of posterior probability accumulated above 0 (positive estimates) or below 0 (negative estimates).

**Table 3:** Estimates of additive genetic ($\sigma_{a}^{2}$), herd/date ($\sigma_{h}^{2}$), and residual ($\sigma_{e}^{2}$) variances, heritability, and herd/date effect of GC reference values based on GC FA profile, and of direct IR and indirect IR predictions ^1^ of methane intensity fresh cheese and cheese solids based on milk FTIR spectra; and additive genetic (r_G_), herd (r_H_), residual (r_E_), and phenotypic (r_P_) correlations between GC reference and IR-predicted methane emission traits ^1^.

Item	Methane Intensity Fresh Cheese:			Methane Intensity Cheese Solids:		
	GC Reference CH_4_/CY_CURD_	Direct IR CH_4_/CY_CURD-IR_	Indirect IR CH_4_/CY_CURD-IR-IR_	GC Reference CH_4_/CY_SOLIDS_	Direct IR CH_4_/CY_SOLIDS-IR_	Indirect IR CH_4_/CY_SOLIDS-IR-IR_
Cow, N	931	931	857	952	952	873
Descriptive statistics:						
Mean	100.1	100.0	99.4	208.5	207.9	206.9
SD	16.4	13.4	14.7	30.8	23.0	27.7
Variances:						
$\sigma_{a}^{2}$	18.7	15.7	27.1	73.1	28.1	46.1
$\sigma_{h}^{2}$	152.4	90.0	76.9	506.9	252.7	268.2
$\sigma_{e}^{2}$	86.0	54.5	100.9	318.6	179.7	393.3
Heritability:						
Mean ^2^	0.178	0.223	0.211	0.187	0.135	0.105
HPD95 ^3^	0.01; 0.34	0.02; 0.41	0.01; 0.40	0.01; 0.38	0.01; 0.29	0.01; 0.21
Herd/date effect:						
Mean ^2^	0.584	0.559	0.373	0.560	0.545	0.376
HPD95 ^3^	0.49; 0.66	0.47; 0.64	0.28; 0.46	0.47; 0.64	0.45; 0.63	0.28; 0.46
Correlations ^4^ with GC-Reference:						
r_P_	-	**0.75**	**0.62**	-	**0.66**	**0.52**
r_G_	-	**0.93**	**0.87**	-	**0.63**	**0.63**
r_H_	-	**0.94**	**0.92**	-	**0.89**	**0.90**
r_E_	-	**0.39**	**0.25**	-	**0.35**	**0.14**

^1^ Heritability defined as intra-herd/date heritability $h^{2}=\;\sigma_{a}^{2}/(\sigma_{a}^{2}+\sigma_{e}^{2})$; herd/date effect defined as${\;h}_{\mathrm{herd}}={\;\sigma }_{h}^{2}/(\sigma_{a}^{2}+\sigma_{h}^{2}+\sigma_{e}^{2})$; Direct FTIR predictions are based on specific calibrations of FTIR milk spectra, whereas indirect ones are based on FTIR predictions of methane intensity milk (see Table 2) and FTIR predictions of percentage yield of fresh cheese and of cheese solids; ^2^ Mean = mean of the marginal posterior distribution; ^3^ HPD95 = bounds of the 95% high posterior density interval; ^4^ Boldface indicates correlations with >95% of posterior probability accumulated above 0 (positive estimates) or below 0 (negative estimates).

**Table 4:** Estimates of additive genetic ($\sigma_{a}^{2}$), herd/date ($\sigma_{h}^{2}$), and residual ($\sigma_{e}^{2}$) variances, heritability, and herd/date effect of GC reference values based on GC FA profile, and of direct IR and indirect IR predictions ^1^ of daily methane production based on milk FTIR spectra; and additive genetic (r_G_), herd (r_H_), residual (r_E_), and phenotypic (r_P_) correlations between GC reference and IR predicted methane emission traits ^1^.

Item	Methane Production (g/d):					
	GC Reference	Direct IR	Measured Milk Yield (dCM) ×		IR Predicted Milk Yield (dCM_IR_) ×	
	dCH_4_	dCH_4-IR_	CH_4_/CM_IR_	CH_4_/CM_FAIR_	CH_4_/CM_IR_	CH_4_/CM_FAIR_
Cows, N	949	949	850	850	850	850
Descriptive statistics:						
Mean	361	358	361	362	358	359
SD	110	67	107	109	70	71
Variances:						
$\sigma_{a}^{2}$	786	412	960	1081	663	741
$\sigma_{h}^{2}$	4679	1519	4542	4729	1547	1649
$\sigma_{e}^{2}$	4762	1679	4358	4529	1571	1631
Heritability:						
Mean ^2^	0.142	0.197	0.180	0.193	0.300	0.312
HPD95 ^3^	0.01; 0.29	0.01; 0.37	0.01; 0.34	0.03; 0.35	0.09; 0.50	0.09; 0.53
Herd/date effect:						
Mean ^2^	0.454	0.418	0.457	0.454	0.406	0.407
HPD95 ^3^	0.36; 0.54	0.32; 0.50	0.36; 0.54	0.36; 0.54	0.31; 0.49	0.31; 0.49
Correlations ^4^ with GC-Reference:						
r_P_	-	**0.54**	**0.95**	**0.95**	**0.53**	**0.55**
r_G_	-	**0.42**	**0.98**	**0.96**	**0.22**	**0.29**
r_H_	-	**0.82**	**0.98**	**0.98**	**0.83**	**0.84**
r_E_	-	**0.31**	**0.91**	**0.92**	**0.31**	**0.34**

^1^ Heritability defined as intra-herd/date heritability $h^{2}=\;\sigma_{a}^{2}/(\sigma_{a}^{2}+\sigma_{e}^{2})$; herd/date effect defined as${\;h}_{\mathrm{herd}}={\;\sigma }_{h}^{2}/(\sigma_{a}^{2}+\sigma_{h}^{2}+\sigma_{e}^{2})$; Direct FTIR predictions are based on specific calibrations of FTIR milk spectra, whereas indirect ones are based on direct (CH_4_/CM_IR_) and indirect (CH_4_/CM_FAIR_) FTIR predictions of the methane intensity milk (see Table 2) multiplied for measured or FTIR predicted daily milk yield; ^2^ Mean = mean of the marginal posterior distribution; ^3^ HPD95 = bounds of the 95% high posterior density interval; ^4^ Boldface indicates correlations with >95% of posterior probability accumulated above 0 (positive estimates) or below 0 (negative estimates).
